# Protection and diagnostic interference induced by heat-inactivated, phage-inactivated and live vaccine prototypes against animal tuberculosis

**DOI:** 10.3389/fvets.2025.1620497

**Published:** 2025-07-21

**Authors:** Leire Fernández-Veiga, Miguel Fuertes, María V. Geijo, Natalia Elguezabal, Jose L. Serrano-Mestre, Lucía Vázquez-Iniesta, Rafael Prados-Rosales, Lorraine Michelet, Maria Laura Boschiroli, Bernat Pérez de Val, Gareth J. Jones, Ramón A. Juste, Joseba M. Garrido, Iker A. Sevilla

**Affiliations:** 1 Departamento de Sanidad Animal, NEIKER-Instituto Vasco de Investigación y Desarrollo Agrario, Basque Research and Technology Alliance (BRTA), Derio, Bizkaia, Spain; 2 Department of Preventive Medicine and Public Health and Microbiology, School of Medicine, Universidad Autónoma de Madrid, Madrid, Spain; 3 Université Paris-Est, Laboratoire de Santé Animale, Unité Zoonoses Bactériennes, Agence nationale de sécurité sanitaire de l'alimentation, de l'environnement et du travail (ANSES), Maisons-Alfort, France; 4 IRTA, Programa de Sanitat Animal, Centre de Recerca en Sanitat Animal (CReSA), Campus de la Universitat Autònoma de Barcelona (UAB), Bellaterra, Catalonia, Spain; 5 Unitat mixta d'investigació IRTA-UAB en Sanitat Animal, CReSA, Campus de la UAB, Bellaterra, Catalonia, Spain; 6 Department of Bacteriology, Animal and Plant Health Agency (APHA), Addlestone, United Kingdom

**Keywords:** animal tuberculosis, vaccine, diagnostic interference, *Mycobacterium bovis*, *Mycobacterium caprae*, *Mycobacterium microti*, phage

## Abstract

**Introduction:**

Vaccination emerges as a promising cost-effective tool to reduce the impact and spread of animal tuberculosis, especially in regions where test-and-slaughter eradication strategy is socioeconomically unfeasible or unfruitful for different reasons, provided it is safe, efficacious and compatible with diagnosis.

**Methods:**

In this study, we preliminarily evaluated the diagnostic interference (using guinea pigs) and the protective efficacy (using mice) of three heat-inactivated, three phage-inactivated and one live attenuated vaccine prototypes prepared from *M. bovis, M. caprae*, and *M. microti*.

**Results and discussion:**

Phage-inactivation killed almost all (96.41–99.92%) bacteria to be included in vaccines and filtering was used to remove the remaining viable cells. All the assayed vaccines induced skin test reactions in response to bovine tuberculin, but they were smaller in the phage-inactivated vaccine groups. All the vaccines were diagnosis-compatible with defined skin test antigens based on ESAT-6, CFP-10, and Rv3615c. In contrast with the rest of prototypes, vaccination with heat- and phage-inactivated *M. microti* did not prompt the production of detectable anti-MPB70+MPB83 antibodies. Mean bacterial burden was lower in all vaccinated groups in comparison with the control, being significantly reduced in the lungs of the heat-inactivated *M. microti* and *M. caprae* and phage-inactivated *M. caprae* groups. Considering both diagnostic interference and protection collectively, the heat-inactivated *M. microti* vaccine showed the best performance. Further studies to evaluate these vaccines and to improve phage-driven inactivation are warranted.

## 1 Introduction

Animal tuberculosis (TB) causes important economic losses in the livestock industry and is a recognized public health issue (zoonosis), especially in middle and low-income regions (1). Originally, bovine TB was thought to be solely caused by *Mycobacterium bovis*, a member of the *Mycobacterium tuberculosis* complex (MTBC). However, any bacteria from the complex can act as a potential causal agent. The main microorganisms responsible for TB in both domestic and wild animals as well as for zoonotic TB in humans are *M. bovis* and *M. caprae* (1–7).

In the EU, the main economic impact of TB lies in the huge costs derived from the application of bovine TB eradication programmes which are mainly based on routine testing and abattoir surveillance in search of TB lesions. The *ante mortem* diagnostic methods for granting and maintaining MTBC infection-free status include the single and comparative intradermal tuberculin tests (SITT and CITT) and the *in vitro* interferon-γ (IFN-γ) release assay (IGRA) (Commission delegated Regulation (EU) 2020/689 of 17 December 2019). In Spain, where goat population is one of the largest in the EU, TB represents a significant health challenge to the goat industry and several regional caprine TB eradication programmes have been implemented (8).

Although eradication programs have made great progress overall, the goal of eradication has not been achieved in several regions even after being in force for decades due to the re-emergence of residual infections, the existence of domestic and wild reservoirs and the limitations of diagnostic tests (9–11). While enhancing the performance of diagnostic techniques as well as the application of hygienic and biosecurity measures is an essential approach, vaccination remains a cost-effective and sustainable method for managing infectious diseases. *M. bovis* Bacille Calmette-Guérin vaccine (BCG) is the only licensed vaccine for TB in humans (in the UK also in badgers). BCG vaccine effectiveness in cattle and goats has been repeatedly demonstrated, but its use in cattle in the field has not been put into practice because of its partial protection and cross-reactivity with TB diagnostic reagents (12), being explicitly forbidden in the EU for the moment. Animal BCG vaccination has additional issues like shelf life and potential persistence of bacilli in animal tissues, products, or environment. Despite this, BCG continued to be an attractive alternative to control bovine TB and thus defined skin test antigens able to differentiate infected from vaccinated animals (DIVA) have been developed and tuned up over time to make BCG vaccination compatible with diagnosis (13). This strategy is being studied in the field in different countries [reviewed in Animal and Plant Health Agency (14)].

The use of inactivated or subunit vaccines can circumvent some of the issues of BCG vaccination (15). Our group developed a heat-inactivated *M. bovis* (HIMB) vaccine (16) that showed promising results in both domestic and wild animals after administration by either oral or parenteral routes and with no or low reactivity with different *in vivo* diagnostic reagents (17–25). The efficacy of parenteral vaccination with HIMB has been shown to be comparable to that of BCG vaccination in goats experimentally challenged with *M. caprae* (17). Regarding vaccination with live attenuated strains other than BCG, *M. microti* (MTBC member) was also used in the past in large-scale trials in humans [reviewed in Orgeur et al. (26)]. The possibility of using different *M. microti* strains for vaccination has regained interest more recently (26–28). Vole-type *M. microti* laboratory strain OV254 and especially the vaccine strain ATCC 35782 (originated from OV166 strain), showed a strongly attenuated virulence even compared to BCG as well as a similar protective efficacy, and thus suggested that the latter could be a clear candidate for vaccination of immunocompromised individuals in whom BCG vaccination is contra-indicated (26). In addition, vaccination with *M. microti* would also be compatible with the DIVA strategy due to genomic deletions of genes coding for antigens included in DIVA reagents (RD1_mic_) as in BCG (RD1_BCG_) (29).

On the other hand, since heat-inactivation can result in uncontrolled rough denaturation of bacterial antigenic compounds with potential immunostimulant effects, we explored the possibility of inactivating mycobacteria through bacteriophages for vaccine development. Mycobacteriophages infect and lyse mycobacteria through dedicated enzymes targeting their complex cell envelope arrangement (30), and thus phage inactivated mycobacteria could keep their antigenic repertoire more intact. In addition, phages are intrinsically capable of stimulating both humoral and cell-mediated arms of the immune system and are considered strong adjuvants (31).

In this study, we evaluated the degree of diagnostic interference and the protective efficacy of three heat-inactivated, three phage-inactivated and one live attenuated vaccine preparations through a preliminary screening in not challenged guinea pigs (to assess diagnostic interference) and challenged mice (to assess protection).

## 2 Methods

### 2.1 Bacteria and mycobacteriophage D29

Table 1 shows the list of the mycobacterial isolates used throughout the study and the use made of them. Bacterial growth for heat-inactivated vaccines, live vaccine, challenge inocula and bacterial whole-cell lysates for immunoblotting, was obtained by culture in Difco™ Middlebrook 7H9 (M7H9) broth (Becton, Dickinson and Company, Sparks, MD, USA) supplemented with 10% BD BBL™ Middlebrook OADC enrichment (Becton, Dickinson and Company), 0.2% glycerol and 0.05% Tween 80 (Sigma-Aldrich, Co. Ltd., Haverhill, United Kingdom). For phage-inactivated vaccines, bacteria were grown in M7H9-OADC (without glycerol and Tween 80). The initial stock of D29 bacteriophage was kindly provided by Prof. Irene Grant (Queen's University Belfast, Belfast, Northern Ireland, UK). D29 was propagated in *Mycobacterium smegmatis* mc^2^155 (ATCC 700084) based on the protocol previously described (32). Briefly, 1 ml of a stationary culture of *M. smegmatis* mc^2^155 with 10^8^ colony forming units (CFU) per ml, 0.1 ml of a D29 phage suspension containing 10^3^-10^4^ plaque forming units (PFU), 5 ml of 1 mM CaCl_2_ M7H9-OADC and 5 ml of molten M7H9 agar (1.5%) (cooled to 55°C) were added to Petri dishes, promptly mixed by gentle swirling, and left solidify for 30 min. After overnight incubation at 37°C, transparent plaques formed on *M. smegmatis* lawn. Plates were flooded with 5 ml of 1 mM CaCl_2_ M7H9-OADC and incubated again overnight at 4°C under gentle orbital shaking. The supernatant medium was recovered and filtered through Minisart 0.2 μm filters (Sartorius Stedim Biotech GmbH, Goettingen, Germany) to get a D29 suspension with 10^10^-10^11^ PFU/ml.

**Table 1:** Mycobacterial strains used throughout the study.

**Species**	**Strain (MTBC spoligotype)**	**Host**	**Use**
*M. bovis*	1403 (SB0339)	Wild boar	Heat- and phage-inactivated vaccine (HIMB and PIMB)
*M. bovis*	2575/08 (SB0339)	Wild boar	Guinea pig sensitization and whole-cell lysate for immunoblotting
*M. caprae*	OVIONE (SB0157)	Sheep	Heat- and phage-inactivated vaccine (HIMC and PIMC)
*M. caprae*	CAT2008 (SB0416)	Goat	Mice challenge, guinea pig sensitization and whole-cell lysate for immunoblotting
*M. microti*	16Z002093 (SB2272)	Badger	Heat- and phage-inactivated vaccine (HIMM and PIMM)
*M. microti*	OV183, ATCC 11152 (SB0118)^a, b^	Vole	Live attenuated vaccine (LMM) and whole-cell lysate for immunoblotting
*M. smegmatis*	mc^2^155, ATCC 700084 (none)^a^	Human	D29 phage amplification

^a^ATCC strain obtained from LGC Standards S.L.U., Barcelona, Spain.

^b^Vaccination with this strain in a pulmonary TB murine model significantly reduced M. tuberculosis H37Rv burden in lungs (28).

### 2.2 Laboratory animals

For experiments to study the diagnostic interference of vaccines, 72 specific pathogen-free Dunkin Hartley (HsdPoc:DH) female guinea pigs weighing 300–349 g were obtained from Envigo (Envigo, Horst, Netherlands). Animals were housed in groups of four (18 groups) in GP-SUITE cage racks (Tecniplast S.p.A., Buguggiate, Varese, Italy). The guinea pig was chosen for this purpose because it is the species to be used in bioassays to assess the safety, sensitizing effect and potency of tuberculins through intradermal skin testing in agreement with the guidelines of the World Organisation for Animal Health (33). It also provided sufficient blood for blood-based assays. To evaluate vaccines' protective effects, 48 specific pathogen-free 6-week-old C57BL/6JOlaHsd female mice were obtained (Envigo) and housed in groups of six (8 groups) in individually ventilated Green Line GM500 cages in racks served by a Smart Flow Air Handling Unit (Tecniplast S.p.A). The mouse intranasal TB model was chosen for the assessment of protection (effects on CFU burden) because it is economic, convenient, highly informative and widely used to study the efficacy of TB vaccines. All animals were placed in the biosafety level 3 animal facilities at NEIKER (Derio, Spain) with *ad libitum* access to water and food and went through a 2-week acclimatization period before the beginning of the experiments. All the experiments involving animals were designed and conducted in accordance with the current three Rs principle and are reported in agreement with the ARRIVE guidelines. The only criteria for inclusion/exclusion determined in advance were sex and weight or age. Allocation of animals into groups was random but no specific randomization method was used. The number of guinea pigs per group needed for experiment A (*n* = 4) was determined with a statistical power of 80% (1 – β = 0.80) and an α risk of 0.05, to detect an increase of 200% in skin reactions considering that skin test negative animals could present mean reactions of as much as 5 mm^2^ (standard deviation = 5 mm^2^). The same number of animals per group was enrolled in experiment B as no previous data was available for guinea pig IGRA and antibody ELISA. The number of mice per group was calculated to detect as statistically significant a difference of 1 Log_10_ in the CFU lung load of animals with treatment compared to animals without treatment, assuming a deviation of 0.6 Log_10_ CFU and having a statistical power percentage of 80% and an α risk of 0.05.

Animal housing, care and experimental procedures were conducted in strict accordance with the regulations of the European, National, and Regional Laws and Ethics Committees that were in force. The experimental design was subjected to ethical review and was approved by NEIKER's Animal Care and Use Committee (NEIKER-OEBA-2020-010) and then authorized by the Department of Agriculture of the competent authority, Bizkaiko Foru Aldundia (2020/52-BFA).

### 2.3 Preparation of vaccines

#### 2.3.1 Heat-inactivated vaccines

Heat-inactivated *M. bovis* (HIMB), *M. caprae* (HIMC) and *M. microti* (HIMM) vaccines preparation was based on a previous report (16). Mycobacteria were grown in the medium specified above for 2–4 weeks. Cells were then harvested by centrifugation (3,000 × g for 10 min) and resuspended in phosphate-buffered saline (PBS) (half the culture volume). Suspensions were sonicated at 37 KHz and below 10°C for 4 min in pulse mode in a Elmasonic P 30H bath (Elma Schmidbauer GmbH, Singen, Germany). Optical density (OD) at 600 nm (OD_600nm_) was adjusted to 1 using PBS. Ten-fold serial dilutions were prepared and plated on agar-solidified M7H9-OADC to estimate CFU numbers per ml. Suspensions were then inactivated at 83–85°C for 45 min in a water bath. Inactivation was confirmed by culture. The inactivated suspensions were adjusted to ~10^8^ CFU/ml according to plate counts and water-in-oil emulsified with Montanide™ ISA 61 VG adjuvant (Seppic, Puteaux, France) in a proportion of 36% antigenic suspension and 64% adjuvant (v/v) using a T 25 digital ULTRA-TURRAX homogenizer equipped with the S 25 N-18 G-ST dispersion tool (IKA, Staufen, Germany). Final concentration of these vaccines was ~3.6 × 10^7^ heat-killed CFU/ml. Vaccine dose volumes were 0.25 ml for guinea pigs and 0.1 ml for mice.

#### 2.3.2 Phage-inactivated vaccines

Mycobacterial growth for phage-inactivated *M. bovis* (PIMB), *M. caprae* (PIMC) and *M. microti* (PIMM) vaccines was obtained after 2 weeks of culture in the medium specified before. After centrifugation, bacterial pellets were resuspended in 2 mM CaCl_2_ M7H9-OADC (without addition of glycerol or Tween 80) and sonicated under the same conditions as for heat-inactivated vaccines. Suspensions' OD_600nm_ were adjusted to 1 using the same medium. One ml was separated to assess pre-treatment CFU concentration by plating serial dilutions. D29 phage (~10^10^ PFU/ml) was added to mycobacterial suspensions at an estimated multiplicity of infection (MOI) of 1:25. Suspensions were left under incubation at 37°C for 30 days with occasional mixing by inversion of culture bottles. Then, after vortexing, an aliquot (1 ml) was removed, washed in 0.05% Tween 80 two times and resuspended in 1 ml. Serial dilutions were plated to estimate post-treatment CFU concentration. Since complete inactivation of mycobacteria was not achieved (see Results), suspensions were filtered first through 5 μm filters and after through 0.2 μm filters (Sartorius) to remove phage attack-resistant cells. Before filtering, *M. bovis, M. caprae* and *M. microti* suspensions had ~8.61 × 10^7^, 2.09 × 10^7^, and 3.44 × 10^7^ phage-killed CFU/ml, respectively. The resulting filtered material was adjuvanted as indicated for heat-inactivated vaccines (36% antigen and 64% adjuvant). Vaccine dose volumes were 0.25 ml for guinea pigs and 0.1 ml for mice.

#### 2.3.3 Live *M. microti* vaccine

*M. microti* OV183 (ATCC 11152) cells were harvested from a 3-week-old culture by centrifugation at 3,000 × g for 10 min. The pellet was weighed as described previously (34) and resuspended to reach a concentration of 0.01 mg/ml. The final doses for guinea pigs and mice were 0.25 ml containing about 2.5 × 10^4^ CFU and 0.1 ml with about 10^4^ CFU, respectively.

### 2.4 Evaluation of vaccine-induced diagnostic interference in guinea pigs

**Figure 1:**
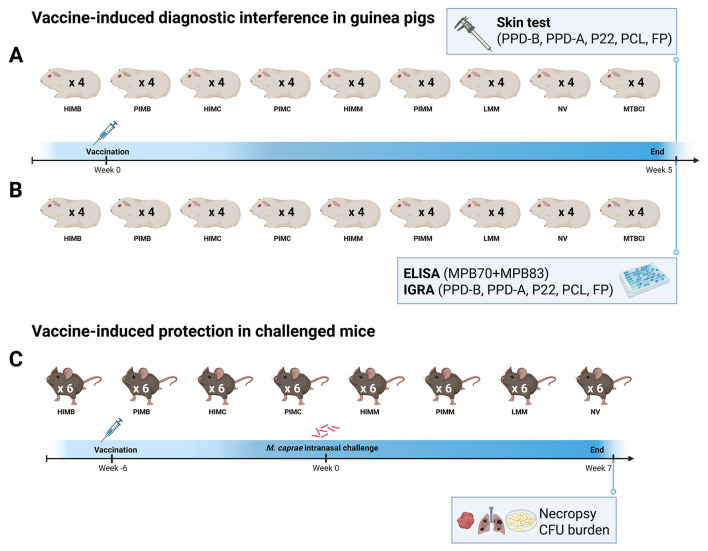
Diagram of the experiments carried out to study vaccine-induced interference in the skin test **(A)** and in ELISA and IGRA **(B)**, and vaccine-induced protection **(C)**. Figure created in BioRender (2025; https://BioRender.com/2wgzesq).

Two sets (A and B) of 8 groups of guinea pigs (4 animals per group) were subcutaneously vaccinated in the inter-scapular region with HIMB, PIMB, HIMC, PIMC, HIMM, PIMM and LMM vaccines or left unvaccinated (not vaccinated control group or NV) (see Figure 1). One additional group per set called MTBC-infected control (MTBCI) was used as reference; two individuals were sensitized with *M. bovis* strain 2575/08 and two with *M. caprae* CAT2008 by intramuscular injection of 0.5 ml saline solution suspensions containing 0.0001 mg (wet weight) bacteria (~10^3^ CFU) as in previous experiments (34).

#### 2.4.1 Skin test (experiment A)

After 5 weeks of vaccination the guinea pigs from set A had their flanks shaved. Skin testing on guinea pigs was performed as described earlier (34). Briefly, each animal was intradermally inoculated (0.1 ml) with official bovine and avian protein purified derivatives (PPD-B and PPD-A) (50 IU each) (CZ Vaccines, O Porriño, Spain) and immunopurified antigenic complex P22 (35) (2 μg) in the left flank, and the defined DIVA antigens that are based on ESAT-6, CFP-10, and Rv3615c (36) peptide cocktail-long (PCL) (1 μg each peptide) (GenScript Biotech, Piscataway, NJ, USA) and triple fusion protein (FP) (3 μg) (36) (Lionex Ltd., Braunschweig, Germany) and saline solution as negative control in the right flank. Antigens were inoculated in a Latin square design per flank with sufficient distance from vaccination site and space between injection sites to avoid potential local immune response interferences. Twenty-four hours after injecting skin test antigens the sites of injections were examined and skin reactions measured with calipers by the same investigator (unaware of groups and antigen positions). The area of erythema (*A*) was calculated using the formula *A* = π × *r*^2^. Afterwards, guinea pigs received deep general anaesthesia with intramuscular xylazine (5 mg/kg; XILAGESIC 2%, Laboratorios Calier SA, Barcelona, Spain) and ketamine (50 mg/kg; Anesketin 100 mg/ml, Dechra Veterinary Products, Barcelona, Spain), and then they were euthanized via intracardiac injection of sodium pentobarbital (200 mg/kg; Dolethal 100 mg/ml, Vetoquinol Especialidades Veterinarias, Madrid, Spain). Following euthanasia, all animals were systematically and thoroughly necropsied. All tissues were macroscopically inspected, and lungs and the nodule formed at the vaccination site underwent mycobacteriological culture. Samples were homogenized in 0.05% Tween 80 (Sigma-Aldrich, Steinheim, Germany) using a GentleMACS™ Dissociator (Miltenyi Biotec, Bergisch Gladbach, Germany) and decontaminated with the BD BBL™ MycoPrep™ kit (Becton, Dickinson and Company, Sparks, MD, USA). The final pellet was inoculated in BBL™ mycobacteria growth indicator tubes (MGIT™) with 10% BACTEC™ MGIT™ 960 supplement kit reagent mixture (BBL™ MGIT™ PANTA™ antibiotics dissolved in BBL™ MGIT™ OADC) (Becton, Dickinson and Company).

#### 2.4.2 IGRA, antibody ELISA and immunoblotting (experiment B)

Five weeks after vaccination, guinea pigs from set B were anesthetized and euthanized like guinea pigs from set A. Whole blood was collected in BD Vacutainer sodium heparin tubes (Becton Dickinson and Company) through cardiac puncture just before euthanasia. Necropsy and tissue sample processing was the same as for experiment A.

**IGRA:** 0.9 ml blood aliquots were distributed into cell culture plate wells. Stimuli (the same used for skin testing) were added to blood aliquots within 2 h of collection at a final assay concentration of 20 μg/ml for PPDs and P22 wells, 1 μg/ml/peptide for PCL and 1 μg/ml for FP. A nil control (PBS) well was also included. Plates were incubated overnight at 37°C in a CO_2_ incubator (5%). After incubation, samples were centrifuged (500 × g, 10 min) and the plasma was collected and stored at −80°C until use. Detection and quantification of the IFN-γ present in plasma was performed using a guinea pig IFN-γ ELISA kit (Cusabio, Wuhan, Hubei, China) following the instructions of the manufacturer. Plates were read at OD_450nm_ and OD_570nm_ using a Multiskan FC spectrophotometer (Thermo Fisher Scientific, Shangai, China). The corrected (OD_450nm − 570*nm*_) mean OD values were used to estimate the IFN-γ concentration according to the curve generated with the standards included in the kit.

**Antibody detection ELISA:** Plasma obtained from non-stimulated sodium heparin blood was analyzed for antibodies against MPB70 and MPB83, which are specific for MTBC (37), using an IgG indirect ELISA adapted from a previous protocol (38). 96-well Nunc-Maxisorp™ plates (Thermo Fisher Scientific, Roskilde, Denmark) were coated with recombinant MPB70 and MPB83 (Lionex GmbH, Braunschweig, Germany) at a final concentration of 0.5 μg/ml each in carbonate/bicarbonate buffer by overnight incubation at 4°C. Each plate included two coated wells and an uncoated well per sample for unspecific background signal subtraction. After washing with PBS containing 0.05% Tween 20 (PBS-T) (Sigma-Aldrich), wells were blocked for 45 min at 37°C with 0.5% casein (Sigma-Aldrich) dissolved in PBS-T. Sera were diluted 1/50 in PBS-T containing 1% casein, loaded in the plate and incubated for 1 h at 37°C. Plates were washed again and 0.1 ml Goat Anti-Guinea Pig IgG Antibody-horse radish peroxidase (HRP) conjugate (Sigma-Aldrich) diluted to 1:5,000 (v/v) was added. After an incubation of 1 h at 37°C, the reaction was developed with 3,3′,5,5′-Tetramethylbenzidine (Sigma-Aldrich) and stopped with H_2_SO_4_. Plates were read (OD_450nm_ and OD_650nm_) in the spectrophotometer. The corrected OD_450nm − 650*nm*_ obtained for each sample was further adjusted by subtracting the value of the uncoated well to the mean value of coated wells. After running the ELISA with plates coated with MPB70 and MPB83 together (MPB70+MPB83 ELISA), samples were re-analyzed using plates coated with MPB70 alone (MPB70 ELISA) and plates coated with MPB83 alone (MPB83 ELISA).

**SDS-PAGE and immunoblotting:** Pooled guinea pig plasma reactivity against bacterial whole-cell lysates was tested by immunoblotting. Mycobacterial growth from *M. bovis, M. caprae* and *M. microti* (Table 1) 3-weeks-old cultures (10 ml) was pelleted and washed with PBS three times. Pellets were resuspended in residual PBS and lysis buffer was added [50 mM Tris-HCl pH 7.5, 150 mM NaCl, 50 mM Dithiothreitol and 1 × protease inhibitor cocktail (Roche)]. The mixture was transferred into tubes containing zirconia/silica beads (0.1 mm). Disruption of bacteria was performed by ten runs of 1 min at 30 Hz in a Tissue Lyser II (Qiagen GmbH, Hilden, Germany). Samples were kept on ice for 1 min between runs. Whole-cell lysates consisted of the supernatant collected after centrifugation of samples at 2,000 × g (4°C) for 10 min. Lysates were resolved by SDS-PAGE and transferred to nitrocellulose membranes and then blocked in PBS with 5% milk and 0.1% Tween 20 overnight. Membranes were put in a blotting frame with individual channels and then incubated overnight at 4°C with plasma samples (diluted 1:200 in blocking buffer). Plasma from the two *M. caprae*-sensitized animals included in the MTBCI was not available for immunoblotting. Channels were washed and then incubated with Goat Anti-Guinea Pig IgG Antibody-HRP conjugate (diluted 1:10,000) (Sigma-Aldrich) for 1 h at room temperature. Reactions were developed using luminol (Pierce). The immunoreactive bands were visualized by autoradiography and reactivity intensity on scanned images was measured and plotted using freely available ImageJ software v2.16.0/1.54p.

#### 2.4.3 Amplification and sequencing of MPB70 and MPB83 genes

To determine if the *M. microti* strain used to prepare HIMM and PIMM (16Z002093; Table 1) contains unmodified MPB70 and MPB83 genes, PCR and sequencing was performed for this *M. microti* strain as well as for other four *M. microti* (with different spoligotypes), one *M. bovis*, one *M. caprae* and one *M. tuberculosis* strains that were used as controls. Amplification was carried out using primers MPB70-F (5′-GGTAGCGAGACGGCACAA-3′) and MPB70-R (5′-GCCCGAACATTCTGGCACA-3′) for MPB70 (694 bp) and MPB83-F (5′-AGGAGGACGGCGTTCAAC-3′) and MPB83-R (5′-GGATGAGCGAGGCAAACC-3′) for MPB83 (765 bp). Reaction was performed in a final volume of 25 μl containing 1x Qiagen PCR buffer, 1x Q-buffer, 200 μM of each dNTPs (Promega, France), 0.5 μM of each primer and 0.5 U of HotStarTaq DNA Polymerase (Qiagen, France). DNA was denatured at 94°C for 15 min, followed by 5 cycles at 94°C for 60 s, 65°C for 30 s, and t 72°C for 2 min, followed by 50 cycles at 94°C for 60 s, 60°C for 60 s, and 72°C for 2 min, plus one cycle at 72°C for 10 min. Purification and Sanger sequencing of amplicons was performed at EuroFins GATC Biotech GmbH (Konstanz, Germany) with the same primers used for amplification.

### 2.5 Evaluation of vaccine-induced protection in challenged mice (experiment C)

Eight mice groups (6 animals each) were subcutaneously vaccinated in the inter-scapular region with the same HIMB, PIMB, HIMC, PIMC, HIMM, PIMM, and LMM vaccines used with guinea pigs, leaving one unvaccinated group (NV group; no treatment) (see Figure 1). Six weeks after immunization, mice were anesthetized with 4% isoflurane (IsoFlo^®^ Zoetis Spain S.L., Madrid, Spain) using a SomnoSuite^®^ Low-Flow Anesthesia System (Kent Scientific Corp, Torrington, CT, USA) and intranasally challenged as described earlier (39). The infective dose consisted of 500 CFU of *M. caprae* CAT2008 suspended in 50 μl saline that was delivered divided into halves onto each nostril. Seven weeks after challenge, all animals were euthanized by CO_2_ administration followed by cervical dislocation. During necropsy, tissues were macroscopically inspected and whole lungs and spleen were removed to determine their bacterial burden. Each organ was homogenized in 5 ml sterile water with 0.05% Tween 80 using a GentleMACS™ Dissociator. CFU load was estimated by plating undiluted homogenates and serial dilutions directly onto M7H9 agar plates containing 10% BACTEC™ MGIT™ 960 supplement and 0.2% glycerol. Since animals in LMM group were vaccinated with live *M. microti*, 5 individual colonies per animal and organ were randomly selected from plates (because colonies displaying the morphology seen previously for this *M. microti* isolate were not present) for standard DVR-spoligotyping (40) and PCR detection of some regions of difference (RD) (41) to confirm they belonged to *M. caprae* challenge strain and not to *M. microti* vaccine. RD detection PCRs were also performed using DNA extracted directly from an aliquot of tissue homogenates (0.125 ml) and positive MGITs (1 ml pelleted) as described earlier (41).

### 2.6 Data analysis

Statistical analysis and data plotting was performed with GraphPad Prism version 10.1.0. Comparisons between the means of skin test response (erythema areas) of every guinea pig group to each antigen and the mean response to the different antigens within the same treatment group were compared using one-way ANOVA with Tukey test for multiple comparisons. Antibody ELISA OD values were also compared using this method. The bacterial loads estimated were compared between mice groups using Kruskal-Wallis test with *post hoc* Dunn's test. The threshold for statistical significance was set at a *p*-value of < 0.05.

## 3 Results

### 3.1 Phage-mediated inactivation of mycobacteria used for vaccines

To assess the degree of inactivation level of *M. bovis, M. caprae*, and *M. microti* strains CFUs were counted before and after phage treatment. Results are shown in Table 2. Phage treatment inactivated between 96.41 and 99.92% of CFUs, depending on the strain. After phage treatment, suspensions contained 3.4 × 10^7^-8.6 × 10^7^ phage-inactivated CFU/ml. The method chosen to remove viable mycobacteria was filter-sterilization.

**Table 2:** Effect of D29 phage treatment on CFU counts of mycobacterial strains.

**Strain**	**Pre-treatment CFU/ml**	**Post-treatment CFU/ml**	**Phage-killed CFU/ml (Pre–Post)**	**CFU/ml reduction %**
*M. bovis* 1403	8.62 × 10^7^	6.93 × 10^4^	8.613 × 10^7^	99.92
*M. caprae* CAT2008	2.09 × 10^7^	4.27 × 10^4^	2.086 × 10^7^	99.80
*M. microti* 16Z002093	3.57 × 10^7^	1.28 × 10^6^	3.442 × 10^7^	96.41

### 3.2 Interference of vaccines on skin test in guinea pigs (experiment A)

**Figure 2:**
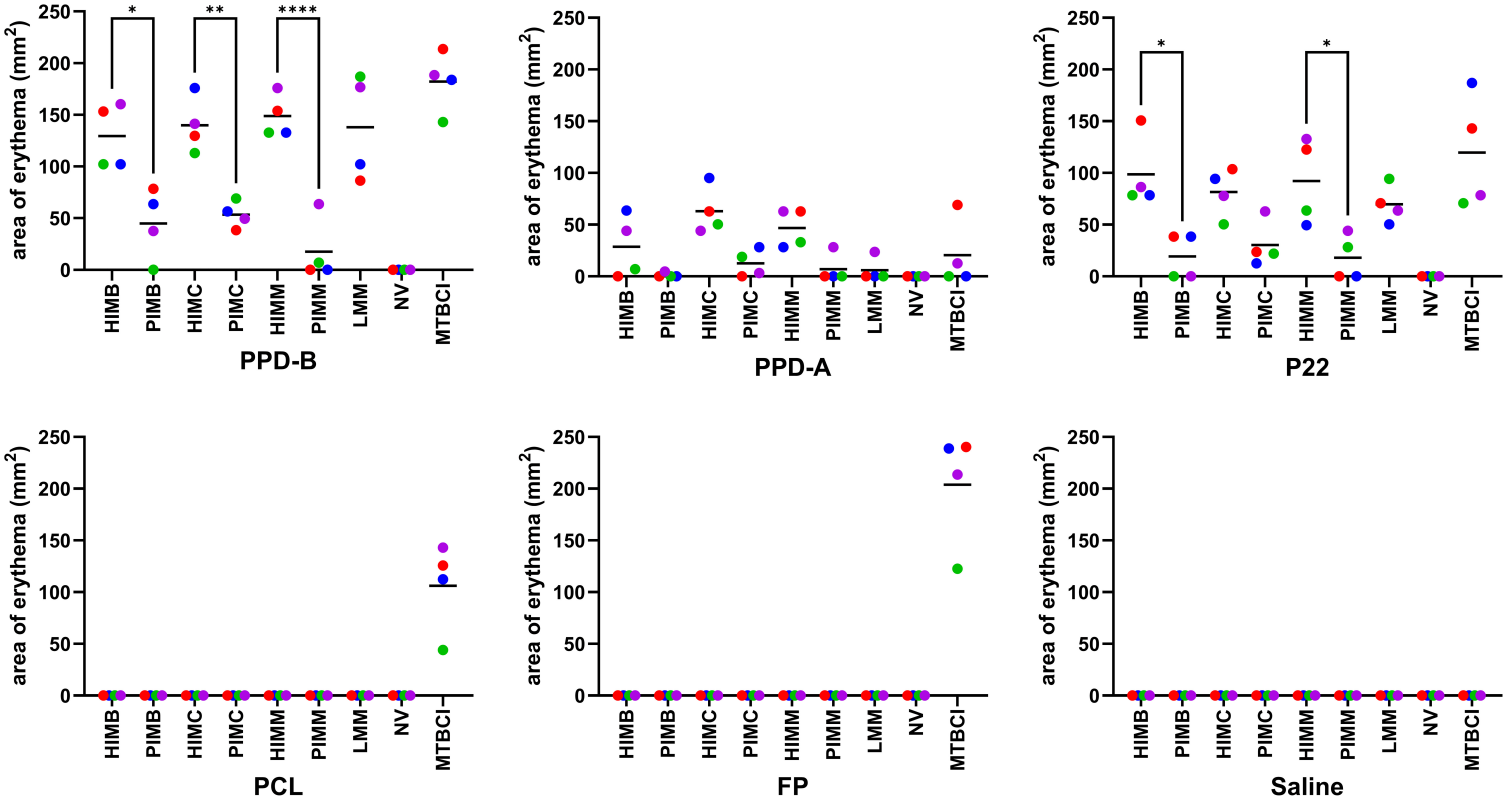
Guinea pig experiment A. Individual (dots) and group mean (horizontal bars) skin test reaction sizes observed in response to the different diagnostic antigens assayed. Abbreviations: HIMB, heat-inactivated *M. bovis*; PIMB, phage-inactivated *M. bovis*; HIMC, heat-inactivated *M. caprae*; PIMC, phage-inactivated *M. caprae*; HIMM, heat-inactivated *M. microti*; PIMM, phage-inactivated *M. microti*; LMM, live *M. microti*; NV, not vaccinated; MTBCI, *M. tuberculosis* complex-infected. One-way ANOVA with *post hoc* Tukey test; statistical significances with 95% confidence interval: *P < 0.05, **P < 0.01, and **** *P* < 0.0001.

Five weeks after vaccination, guinea pigs from experiment A were skin tested. The erythema (inflammatory response) areas at the sites of inoculation of antigens observed at 24 h are shown in Figure 2. As expected, guinea pigs from the NV control group did not show any reactions to any of the skin test antigens used. Conversely, all MTBCI group individuals reacted to all MTBC skin test antigens.

All individuals from heat-inactivated vaccine groups (HIMB, HIMC and HIMM) developed skin reactions to PPD-B that were not significantly different from those displayed by MTBCI control animals. In contrast, the response of guinea pigs from phage-inactivated vaccine groups was significantly lower not only in comparison with MTBCI group but also in comparison with their homologue heat-inactivated vaccine groups (see Figure 2). Although reactions were smaller in general and HIMC response was not significantly different from PIMC, the outcome for P22 antigen was very similar to that of PPD-B. LMM vaccination caused similar responses to both PPD-B and P22 that were statistically undistinguishable from any heat-inactivated vaccine and MTBCI groups. Mean skin erythema areas to PPD-A were reduced in general, being significantly smaller than PPD-B areas in all groups except in PIMM group (and NV group) (data not shown). However, P22 reactions were only consistently larger than PPD-A reactions for HIMB, LMM and MTBCI. Visible reactions to PCL and FP reagents were only observed in MTBCI guinea pigs, being of a similar magnitude to that obtained for PPD-B. Thus, none of the vaccines assayed did trigger any detectable responses to the inoculation of these defined skin test antigens.

All cultures were negative, including guinea pigs from LMM group that were vaccinated with live mycobacteria (*M. microti*).

### 3.3 Interference of vaccines on IGRA and antibody ELISA in guinea pigs (experiment B)

#### 3.3.1 IGRA

The results of guinea pig blood stimulation followed by detection and quantification of released IFN-γ using the sandwich ELISA kit did not yield reliable results (data not shown). Plate validation was very challenging, and no significant differences were identified amongst validated results, including non-stimulated blood samples. Since the goal of the study was not to validate neither the guinea pig blood stimulation protocol nor the sandwich ELISA kit, repeating experiment B using new guinea pigs and a different IFN-γ quantification ELISA kit was not considered.

#### 3.3.2 Antibody ELISA

**Figure 3:**
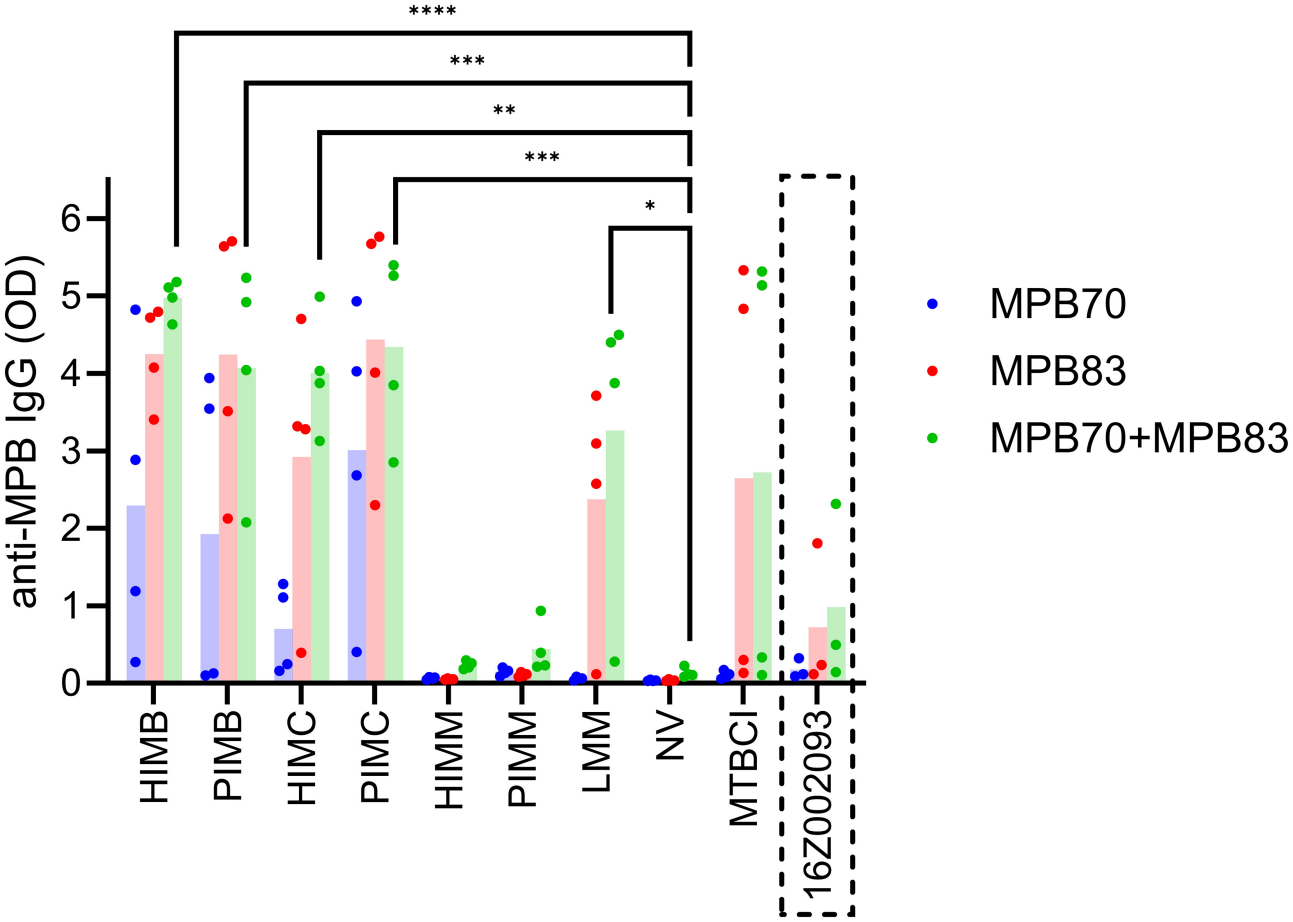
Guinea pig experiment B, antibody ELISA. Individual (dots) and group mean (columns) anti-MPB70+MPB83 IgG antibody titers detected in guinea pigs' plasma after 5 weeks of vaccination. The results for separate MPB70 ELISA and MPB83 ELISA tests are also included. The antibody titers of three guinea pigs from a previous study (34) that were sensitized with live bacteria of the same strain used to prepare HIMM and PIMM (*M. microti* 16Z002093) are included in the last position (inside the discontinuous rectangle). HIMB, heat-inactivated *M. bovis*; PIMB, phage-inactivated *M. bovis*; HIMC, heat-inactivated *M. caprae*; PIMC, phage-inactivated *M. caprae*; HIMM, heat-inactivated *M. microti*; PIMM, phage-inactivated *M. microti*; LMM, live *M. microti*; NV, not vaccinated; MTBCI, *M. tuberculosis* complex-infected. One-way ANOVA with *post hoc* Tukey test; statistical significances with 95% confidence interval, calculated only for MPB70+MPB83 ELISA: *P < 0.05, **P < 0.01, ***P < 0.001, and ****P < 0.0001.

Anti-MPB70+MPB83 IgG antibody titers were measured by ELISA 5 weeks after vaccination and results are shown in Figure 3. Most tested vaccines induced a humoral response as revealed by enhanced production of antibodies against MPB70+MPB83 antigenic mixture: both variants of vaccines based on *M. bovis* and *M. caprae* (HIMB, PIMB, HIMC, PIMC) as well as the live vaccine (LMM; prepared using *M. microti* OV183, ATCC 11152; see Table 1) triggered the production of significant antibody titers in comparison with basal levels seen in NV group. In contrast, animals immunized with inactivated vaccines HIMM and PIMM (prepared using *M. microti* 16Z002093 field strain; see Table 1) did not develop anti-MPB70+MPB83 IgG levels different from those seen in non-vaccinated animals. Some individuals from groups vaccinated or sensitized with live mycobacteria (1/4 LMM and 2/4 MTBCI guinea pigs) did not display antibodies against MPB70+MPB83 mixture in their plasma samples according to ELISA results, but the rest of individuals of these groups had antibody titers within the range observed in the other groups displaying antibodies. In view of the disparate results observed for inactivated (HIMM and PIMM) and live (LMM) *M. microti*-based vaccines and taking into account that a different strain was used for inactivated and live preparations, additional tests were performed: first, the *MPB*70 and *MPB83* genes of *M. microti* 16Z002093 field strain were amplified and sequenced, and second, guinea pig sera from animals sensitized with live bacteria of the same strain from a previous study (34) were also analyzed through this antibody ELISA. We observed that *MPB*70 and *MPB83* genes with sequences identical to GenBank EU683971.1 and EU683972.1 sequences of *M. bovis* were present in the strain, and that one out of three sensitized animals developed detectable anti-MPB70+MPB83 antibodies (Figure 3). When anti-MPB70 and anti-MPB83 antibody titers were compared using separate ELISA assays (MPB70 ELISA and MPB83 ELISA), these were always higher for MPB83 than for MPB70. In agreement with MPB70+MPB83 ELISA results, antibodies against individual proteins were not observed for HIMM and PIMM groups. Anti-MPB70 IgG were not detected in the plasmas of guinea pigs vaccinated or sensitized/infected with live mycobacteria.

#### 3.3.3 Immunoblotting

**Figure 4:**
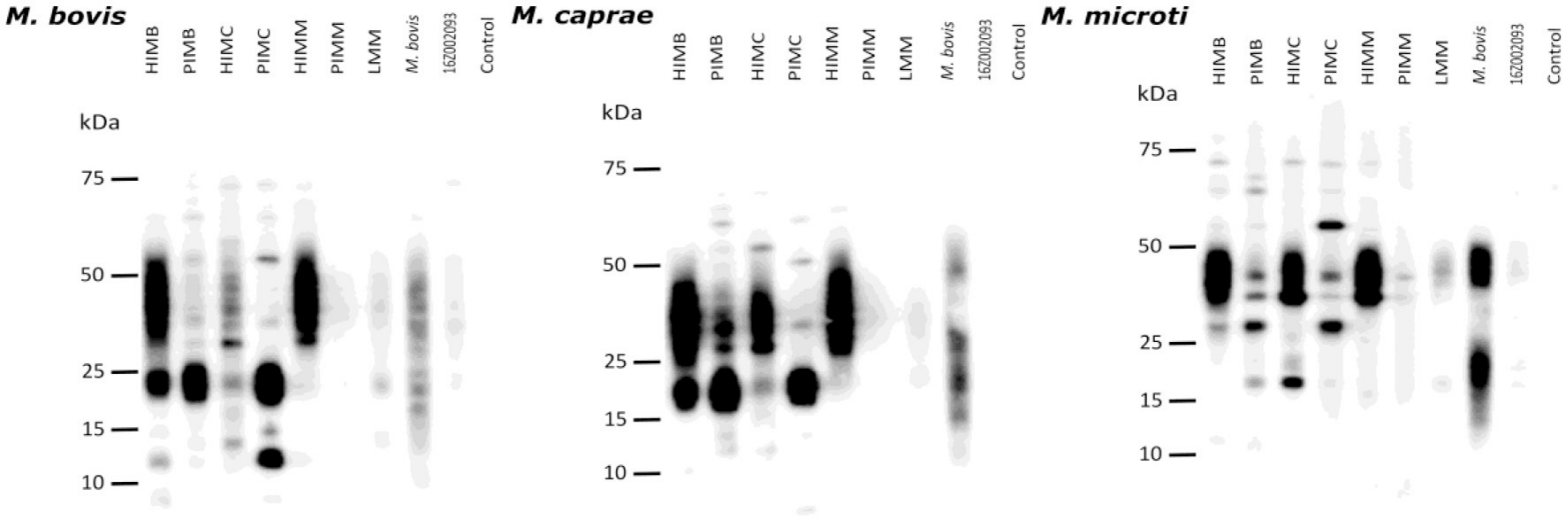
Guinea pig experiment B, mycobacterial whole-cell lysate immunoblots. *M. bovis* **(left)**, *M. caprae* **(center)** and *M. microti* **(right)** lysates were separated by SDS-PAGE and transferred to membranes. Reactivity with the plasmas from different guinea pig groups was revealed by Western-blotting. Groups: heat-inactivated *M. bovis* (HIMB); phage-inactivated *M. bovis* (PIMB); heat-inactivated *M. caprae* (HIMC); phage-inactivated *M. caprae* (PIMC); heat-inactivated *M. microti* (HIMM); phage-inactivated *M. microti* (PIMM); Live *M. microti* vaccinated (LMM); *M. bovis*-infected (part of MTBCI); *M. microti*-infected (strain 16Z002093) from a previous experiment (34); non-vaccinated non-infected control.

The immunogenicity induced by vaccination was also assessed by SDS-PAGE and Western-Blotting of whole-cell lysates using the same plasma and the same conjugate as for antibody ELISA. Autoradiographic photographs of immunoblots are displayed in Figure 4 and the plots with the band intensity analysis are included as supplementary material (Supplementary Figure 1). Plasma from guinea pig groups vaccinated with inactivated vaccines showed the strongest reactivities, except for PIMM. Reactivities for antigens ranging between 25 and 55 KDa were much more intense for HI vaccinates than for PI vaccinates. In contrast, PI vaccinates showed stronger signals than HI vaccinates for antigens in the range 20–25 KDa. The signal observed for *M. bovis*-sensitized guinea pigs (from MTBCI) was more evident than that seen for LMM-vaccinated (OV183) or *M. microti*-sensitized (16Z002093) and was similarly distributed along the molecular weight range in comparison to other vaccinated groups, except for *M. microti* lysate.

### 3.4 Protection of vaccines in challenged mice (experiment C)

We next studied the protective efficacy of the vaccines under study by measuring their capacity to restrict the growth of *M. caprae* in the lungs and spleens of challenged mice. One HIMB-immunized mouse showing apathy and abnormal postures (hunched abdomen, tilted head) and one PIMB-immunized mouse with important teeth malformation and malocclusion were euthanized at different time points between vaccination and infective challenge. The necropsy showed a severe bilateral nephrosis in the former that was not considered to be related with the procedures of the experiment. The intranasal bacterial challenge failed to achieve infection in 8 out of the 52 (15%) animals that completed the experiment as assessed by culture and pathological methods and were discarded from further statistical analysis. In summary, CFU counts obtained for three HIMB, four PIMB, five HIMC, four PIMC, six HIMM, six PIMM, six LMM and five NV individuals were used for analysis.

**Figure 5:**
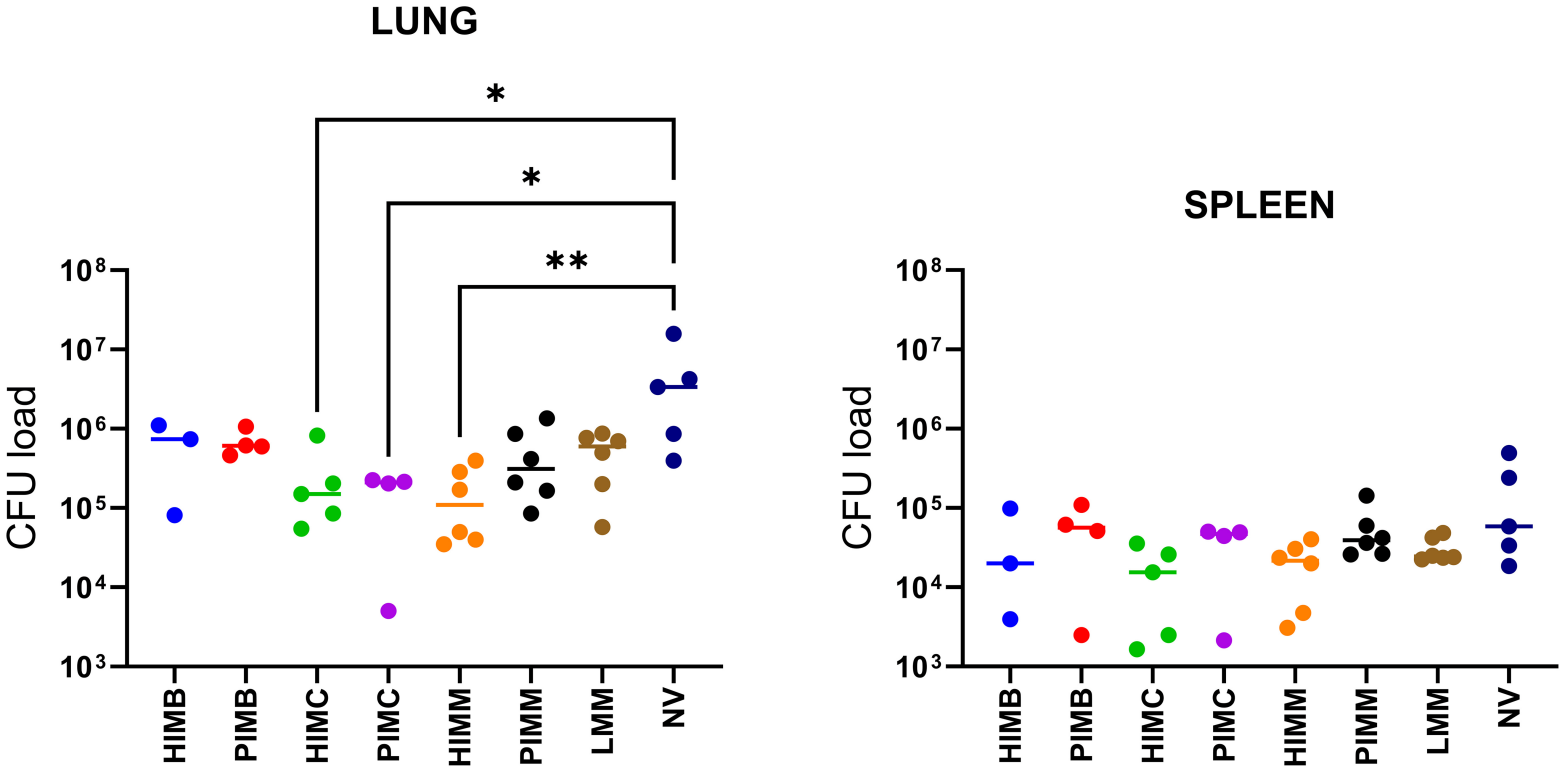
Individual (dots) and group median (horizontal bars) bacterial load (CFU) in the lungs and spleens of mice from experiment C. HIMB, heat-inactivated *M. bovis*; PIMB, phage-inactivated *M. bovis*; HIMC, heat-inactivated *M. caprae*; PIMC, phage-inactivated *M. caprae*; HIMM, heat-inactivated *M. microti*; PIMM, phage-inactivated *M. microti*; LMM, live *M. microti*; NV, not vaccinated. Kruskal-Wallis test with *post hoc* Dunn's test; statistical significances with 95% confidence interval: *P < 0.05 and **P < 0.01.

Lung and spleen bacterial loads are shown in Figure 5. Overall, mean and median CFU values were lower in vaccinated groups relative to NV group. This reduction was significant in the lungs of HIMC (*p* = 0.044), PIMC (*p* = 0.048) and HIMM (*p* = 0.005) groups. Although a similar general trend was observed in the spleens of vaccinated animals, no significant differences were demonstrated in comparison with the NV group. The bacterial loads were higher in lungs than in spleens. When CFU load was computed together for lungs and spleens, the mean of HIMC (*p* = 0.042) and HIMM (*p* = 0.006) groups still differed significantly from that of NV animals.

## 4 Discussion

Despite the huge investments and efforts directed to bovine TB eradication programs and their big achievements, the disease still represents a major challenge in many regions. It is not only of great concern in relation to bovines. Animal TB's impact extends to other domestic (goats, swine, sheep) and wild animals (wild bar, deer, badger etc.). The ability of *M. bovis* and *M. caprae* to infect and be transmitted within and between many of these species as well as its zoonotic nature (33), makes using all available tools against the disease imperative. Vaccination represents a very interesting strategy that could help in reducing the impact of animal TB (15, 42–46), even though most studies have focused on the use of BCG. Inactivated vaccines have several advantages related to viability, safety and stability in comparison to live vaccines (47). Our group developed a HIMB vaccine (16) that has been evaluated by oral and parenteral administration in different animal species with promising results and potential for improvement (15). To minimize to some extent the antigenic denaturation caused by heat killing, we explored the possibility of inactivating mycobacteria using mycobacteriophages. In this study, three heat-inactivated vaccines (including our reference HIMB), three phage-inactivated vaccines and one live vaccine were prepared and parenterally administered to guinea pigs and mice for a preliminary evaluation of their effects in terms of diagnostic interference and protection.

Total inactivation of mycobacteria through D29 phage treatment was not achieved under the conditions used in this study. Nevertheless, we wanted to use these phage-treated mycobacterial suspensions as a proof of concept of phage-inactivated vaccines. To make sure viable cells would not be present in final preparations, phage-treated suspensions were filter-sterilized prior to being inoculated in animals. Different approaches to achieve complete phage-inactivation of bacterial cells or to remove viable cells from phage-treated suspensions are being now studied in an ongoing new research project. For example, using different wild type or mutant mycobacteriophages (independently, sequentially or in a cocktail) (48).

All heat-inactivated vaccines interfered with PPD-B and P22 skin testing, which is consistent with skin test and IGRA results from previous studies in cattle and goats parenterally vaccinated with HIMB (17, 20, 25). In contrast, orally administered HIMB does not cause any interferences in diverse species (15), but this or other mucosal routes were not assessed in the present study. The skin reactions caused by PPD-B and P22 in phage-inactivated vaccination groups were significantly smaller than those seen in heat-inactivated vaccination groups (Figure 2). This difference may be attributed to a possible reduction in the antigenic content of phage-inactivated suspensions after filtering (retained by the filter) in comparison with heat-treated suspension.

None of the vaccines assayed induced intradermal responses to ESAT-6/CFP-10/Rv3615c-based antigenic reagents (PCL and FP) in the skin test. These DIVA diagnostic reagents were developed to differentiate between BCG-vaccinated and MTBC-infected individuals. Our results showed that these antigens would also serve to differentiate between vaccination with these inactivated vaccines (either heat-inactivated or phage-inactivated) and infection. In this sense, previous studies in goats also showed that vaccination with HIMB and HIMC did not cause interferences in ESAT-6/CFP-10 IGRA (17, 21). However, some positivity was observed in a recent study using ESAT-6/CFP-10/Rv3615c IGRA (49). Thanks to deletions (RD1_mic_) like those of BCG (RD1_BCG_), *M. microti*-based live or inactivated vaccines would also be diagnosis-compatible using PCL or FP antigens, as indicated these and previous results (34). The absence of a response to ESAT-6/CFP-10/Rv3615c in guinea pigs vaccinated with the inactivated vaccines could be a consequence of bacterial pellet washing; culture is centrifuged and the medium where mycobacteria have grown is discarded before the inactivation treatment. This seems clear for heat-inactivated vaccines because bacteria are washed and resuspended in PBS and immediately heat-treated. For phage-inactivated vaccines, culture medium is substituted with new medium and re-incubated (30 extra days), but this medium contains both 2 mM CaCl_2_ and phages. It can be speculated that these factors might be able to alter the production or secretion dynamics of those mycobacterial antigens, but we did not perform any experiments to assess this. Regulation of genes involved in the expression of these virulence-associated antigens is very complex and depends, for example, in stimuli altering divalent metal homeostasis and proteostasis (50, 51). Also, mycobacteriophage genomes encode genes capable of influencing host physiology (52).

The ELISA test used with guinea pigs' sera showed that all vaccines except for HIMM and PIMM induced anti-MPB70+MPB83 IgG antibodies production and would cause interference in this immunoassay. This is in line with the results obtained earlier in wild boar, goats, cattle, and rabbits parenterally vaccinated with HIMB (16, 17, 25, 53). Amongst guinea pigs vaccinated with (LMM) or exposed to (MTBCI) live mycobacteria, some of them had detectable antibodies as well (3 out of 4 in LMM group, and one *M. caprae*- and one *M. bovis*-challenged individuals from MTBCI group), indicating that some of them had sufficient time to seroconvert after exposure (5 weeks), while others did not. Anti-MPB70+MPB83 IgG response was boosted in animals vaccinated with inactivated *M. bovis* and *M. caprae* in comparison to animals infected with the same agents. This can be attributed to a potentiation effect of the adjuvant, to a larger bacterial dose in inactivated vaccines than in sensitizing/challenge inocula as well as to a differential induction of the immune response by live or dead mycobacteria. Sera were re-analyzed with separate MPB70 ELISA and MPB83 ELISA. The dominance of MPB83 reactivity over MPB70 reactivity was evident for all groups with detectable anti-MPB70+MPB83 IgGs. Both heat-inactivated and phage-inactivated vaccines (HIMB, HIMC, PIMB and PIMC) stimulated anti-MPB83 antibody production at a similar level. This demonstrated that sufficient MPB83 remained in phage-inactivated vaccines after filtering the phage-treated suspensions. MPB83 can be found in its lipoprotein form associated with the bacterial cell envelope and in its non-lipoprotein form in the culture filtrate, while MPB70 is a highly soluble secreted protein abundantly present in culture filtrates (37). Interestingly, seroreactivity to MPB70 was not observed in guinea pigs inoculated (vaccine or challenge) with live mycobacteria, which is consistent with previous observations indicating that antibodies against MPB70 develop 18 weeks after experimental infection in cattle (54). Mycobacterial whole-cell lysate immunoblots showed a different reactivity pattern for heat-inactivated and phage-inactivated vaccinated groups. Antigen recognition appeared concentrated at different KDa ranges depending on the vaccine inactivation type, while plasma from *M. bovis*-sensitized group displayed a more homogeneous pattern in general. It is reasonable to speculate that these differences are due to a different antigenic profile resulting from heat treatment (denaturation), and phage (phage enzymes cleaving and cutting) and filtering treatments (loss of antigens), but we did not perform further analyses to assess this. For example, Mb1043, HspX, and TB15.3 fall within the 15–25 KDa range, while Ag85, PstS1, and PPE1 are expected within the 25–50 KDa range. It would be worthwhile to test if a vaccine including material from both inactivation methods could provide an enhanced protection relative to those including only either heat- or phage-inactivated preparations.

Regarding the differential humoral response seen between inactivated (HIMM and PIMM) and live (LMM) *M. microti*-based vaccines, we initially hypothesized that the strain used for inactivated vaccines could have genomic deletions or a differential expression of MPB70+MPB83 products in comparison to the one used for LMM vaccine. However, on the one hand, we found no difference in the sequences of these genes, and on the other, one out of the three guinea pigs sensitized with the same strain used for HIMM and PIMM vaccines from a previous study (34) (see Figure 3) did develop low but still detectable IgGs. These results suggest that this *M. microti* strain is indeed able to produce MPB83 at least when infecting animal tissues. Similar differences between mycobacterial species, strains and situation (*in vitro* culture or *in vivo* infection) have been reported previously (37, 55). The differential production, expression and/or secretion of these antigens have been linked with regulation through SigK-RskA system in response to external stimuli (56). The existence of potential genetic variations of SigK or RskA that could explain this difference were not examined. In terms of protection, the effectiveness and actual role of humoral immunity in response to mycobacterial infections is not clear yet (57). It is generally accepted that tuberculosis protective immunity is primarily mediated by cellular immune responses. Despite this, it has been suggested that certain antibody specificities against bacterial surface epitopes and with the correct isotype may confer protection against intracellular infections (37). In this sense, some authors reported higher susceptibility to TB in B-cell deficient mice and prolonged survival of mice infected with an anti-MPB83 antibody-coated virulent *M. bovis* strain (37). In addition, B cells are not just antibody producing cells, they also have active roles as antigen presentation, cytokine production and modulation of T cell responses (57).

To assess the protective ability of vaccines, isoflurane-anesthetized mice were intranasally challenged with virulent *M. caprae*, which was delivered using a micro-pipette in agreement with a previous report (39). Some of the mice (15%) did not aspirate the inoculum and did not get infected. Intranasal challenge with 500 CFU was unfruitful in 10% of mice in the original report as well (39). Unfortunately, our reference vaccine group (HIMB) ended up with only three informative mice out of the six that were initially enrolled; one had to be euthanized, and infection was not achieved in two. Notwithstanding, a reduced bacterial load was seen in all vaccinated groups when compared to non-vaccinated control group (NV). HIMB parenteral vaccination of wild boar and goats has been shown to reduce the bacterial load in thoracic tissues (16, 17). In the present study, the reduction was particularly evident and significant in the lung loads of HIMC, PIMC and HIMM vaccinated groups. This is important because airways are considered to be the most likely way of bacterial shedding and infection dissemination to susceptible populations. The increased effectiveness for *M. caprae*-based vaccines in comparison with *M. bovis*-based vaccines could perhaps be explained by the similarity of the vaccine strain with the infective strain (both are *M. caprae*) as was observed in goats vaccinated with a *M. caprae* autovaccine in relation to goats vaccinated with HIMB (21). Despite this, the ability to limit the bacterial load in mice tissues was collectively the greatest in HIMM vaccinated individuals. Differences might have been greater if the time left after challenge would have been of 4 weeks instead of 7 weeks (39).

Although new questions and challenges have arisen, we have established the basis for the development of phage-inactivated vaccines, and preliminarily characterized the diagnostic interference and protective efficacy of phage-inactivated first prototype vaccines, new heat-inactivated vaccines and a live attenuated vaccine alternative to BCG based on vole-type *M. microti*. A method for complete phage-driven bacterial inactivation is desirable, since resulting vaccines would be expected to be diagnosis-compatible with the same DIVA reagents and protective. Amongst other findings, of note is that HIMM vaccine has emerged as an interesting candidate because it did not interfere with cellular immunity-based diagnosis using ESAT-6/CFP-10/Rv3615c-derived antigens or with humoral immunity-based diagnosis using MPB70+MPB83 antigens, while showing the highest degree of protection. Further research is needed to evaluate these vaccines and to improve phage-driven inactivation to develop new phage-inactivated vaccines.

## Data Availability

The original contributions presented in the study are included in the article/Supplementary material, further inquiries can be directed to the corresponding author.

## Supplementary material

Supplementary Figure 1 Guinea pig experiment B, signal intensity of mycobacterial whole-cell lysate immunoblots. Analysis of the intensity of bands (recognized antigens) in the immunoblots of *M. bovis* (a), *M. caprae* (b), and *M. microti* (c) cell lysates by molecular mass (KDa) range. Groups: HIMB, heat-inactivated M. bovis; PIMB, phage-inactivated *M. bovis*; HIMC, heat-inactivated *M. caprae*; PIMC, phage-inactivated *M. caprae*; HIMM, heat-inactivated *M. microti*; PIMM, phage-inactivated *M. microti*; LMM, live *M. microti* vaccinated (LMM); *M. bovis*-infected (from MTBCI); *M. microti*-infected (strain 16Z002093) from a previous experiment (34); NV, non-vaccinated non-infected control.
