# Supplementary figures and images for: Protection and diagnostic interference induced by heat-inactivated, phage-inactivated and live vaccine prototypes against animal tuberculosis
Source: Front Vet Sci. 2025 Jul 21;12:1620497. doi: 10.3389/fvets.2025.1620497 (PMC12320537; doi:10.3389/fvets.2025.1620497)

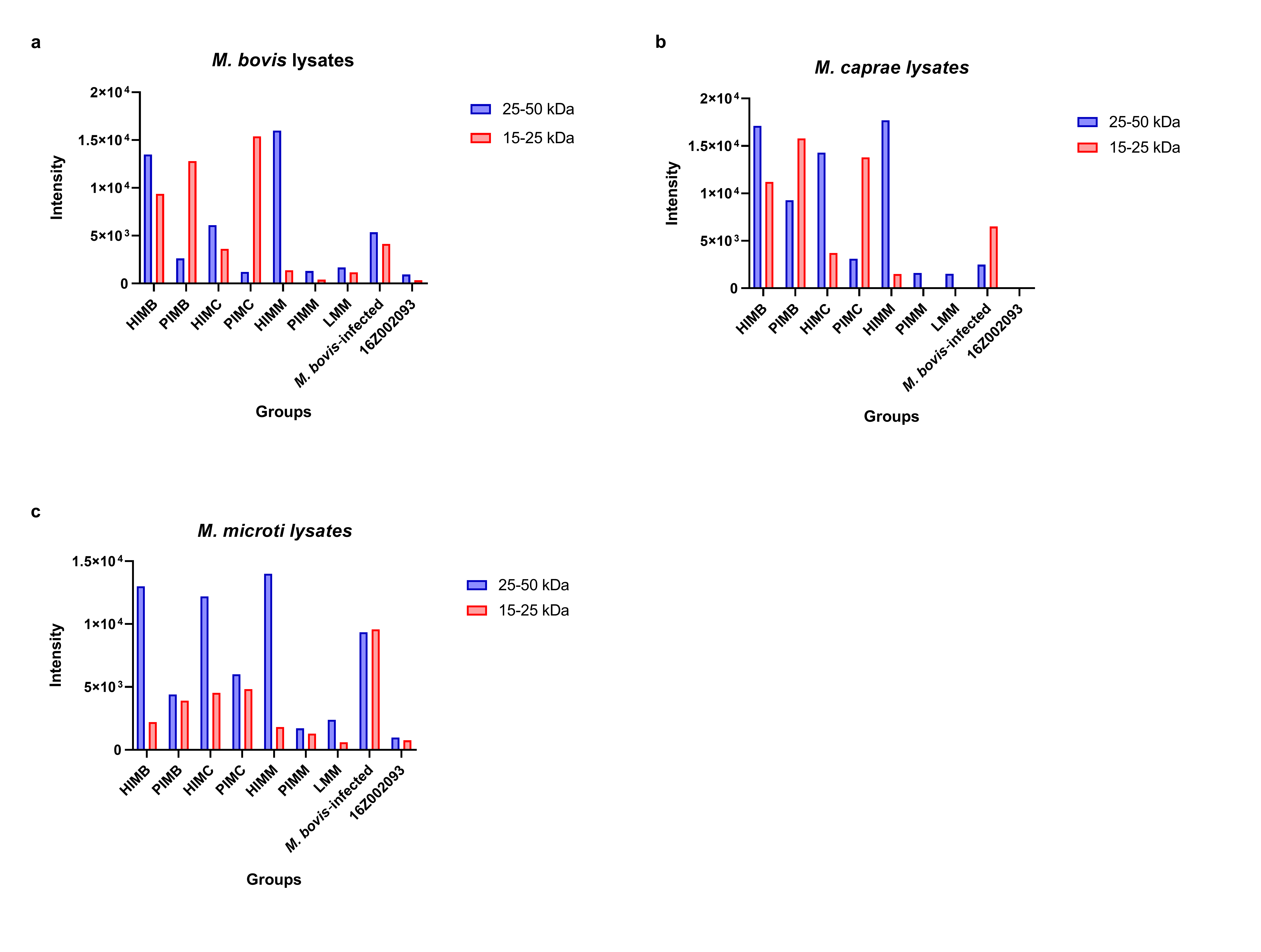

Supplement: Supplementary Figure 1 — Guinea pig experiment B, signal intensity of mycobacterial whole-cell lysate immunoblots. Analysis of the intensity of bands (recognized antigens) in the immunoblots of M. bovis (a), M. caprae (b), and M. microti (c) cell lysates by molecular mass (KDa) range. Groups: HIMB, heat-inactivated M. bovis; PIMB, phage-inactivated M. bovis; HIMC, heat-inactivated M. caprae; PIMC, phage-inactivated M. caprae; HIMM, heat-inactivated M. microti; PIMM, phage-inactivated M. microti; LMM, live M. microti vaccinated (LMM); M. bovis-infected (from MTBCI); M. microti-infected (strain 16Z002093) from a previous experiment (34); NV, non-vaccinated non-infected control. [file Image_1.tif]
